# Characterization of the complete chloroplast genome sequence of *Rubus rufus* Focke (Rosaceae)

**DOI:** 10.1080/23802359.2021.1967810

**Published:** 2021-09-30

**Authors:** Weicheng Huang, Fei Qiao, Wei Guo, Wei Wu

**Affiliations:** Department of Horticulture and Landscape Architecture, Zhongkai University of Agriculture and Engineering, Guangzhou, China

**Keywords:** *Rubus fufus*, Rosaceae, complete chloroplast genome, automated assembly

## Abstract

*Rubus rufus* Focke is a deciduous shrub species native to subtropical China and is valuable resources for the Raspberry cultivation by virtue of prolific fruits and stout resistances to hash stress. In this study, we assembled and characterized the complete chloroplast genome of *R. rufus* as resources for the future study. The chloroplast genome was 156,232 bp in length, composing of one large single copy (LSC; 85,840 bp) and one small single copy (SSC; 18,850 bp), separated by two inverted repeats (IRs; 25,771 bp). A total of 130 genes were predicted, including eight rRNAs, 36 tRNAs, and 86 protein-coding genes. The phylogenetic analysis showed a close relationship between *R. rufus* and *Rubus cochinchinensis* in the tribe Rubeae.

*Rubus rufus* Focke is a climbing shrub native to subtropical China. This species shows great morphological variations, and three varieties have been recognized so far (Lu and Boufford 2003). With prolific fruits and stout resistances to diverse hash stress (Personal observation), *R. rufus* holds great potential for Raspberry cultivation in the future. However, the paucity of genetic resources has hindered the studies on phylogeny, diversity and speciation for this species. Here, we assembled and characterized the complete chloroplast genome sequence of *R. rufus* as a resource for future studies on this species.

We sampled leaves of *R. rufus* var*. rufus* from a forest understory at the Dawei Mountain in Yunnan Province (22°75' N, 103°45' E, alt. 750 m). A voucher specimen was also deposited at the Sun Yat-sen University Herbarium (SYS) with accession number RCOC-18274-XN05 (Weicheng Huang, huangwc0921@163.com). Total genomic DNA was extracted from silica gel–dried leaves using a modified CTAB protocol (Doyle 1991). Using the qualified DNA, a library with insertion size of 500 bp was constructed and sequenced with paired-end (150 bp) read on an Illumina Hiseq 3000 platform (San Diego, CA, USA) at Jierui Bio-Technique Co. Ltd (Beijing, China). Prior to the chloroplast genome assembly, all raw reads were filtered and trimmed with default parameters using the package fastp v0.20.1 (Chen et al. 2018). Then, the clean reads were mapped to the published chloroplast genome of *Rubus leucanthus* (MK105853.1) using BWA v0.7.17 (Li and Durbin 2009), and the obtained reads were fed to package Unicycler v0.4.8 (Wick et al. 2017) for chloroplast genome assembly. Next, the obtained contigs were oriented by aligning to *R. leucanthus* chloroplast genome using Mummer 3.2 (Kurtz et al. 2004). Finally, the genome assemblies were annotated using package Geseq (Tillich et al. 2017) and corrected manually using Geneious v.9.0.2 (Kearse et al. 2012). The complete chloroplast genome sequence of *R. rufus* was submitted to GenBank with the accession number of MW930397.

The complete chloroplast genome of *R. rufus* was 156,232 bp in length, with an 85,840 bp large single copy (LSC) and a 18,850 bp small single copy separated by two inverted repeats (25,771 bp). The chloroplast genome circle is joined end to end in the order of 'LSC-IRA-SSC-IRB'. The overall GC content is 37.18%, A total of 130 genes were predicted, consist of 86 protein-coding genes, 36 tRNA genes and eight rRNA genes.

For phylogenetic analysis, the chloroplast genomes of *R. fufus*, 19 other Roseaceae species, and one outgroup species *Cucumis sativus* (DQ119058.1) were downloaded from GenBank. All the sequences were aligned using MAFFT v.7.313 (Katoh and Standley 2013) and a maximum-likelihood phylogenetic analysis was implemented using RAxML v.8.2.11 (Stamatakis 2014) (Figure 1). The phylogeny analysis demonstrated that *R. rufus* was placed in the Rubeae tribe with close relationship with *Rubus cochinchinensis*, and Rubeae tribe was monophyly with high support for among the five tribes used in this study.

**Figure 1.**
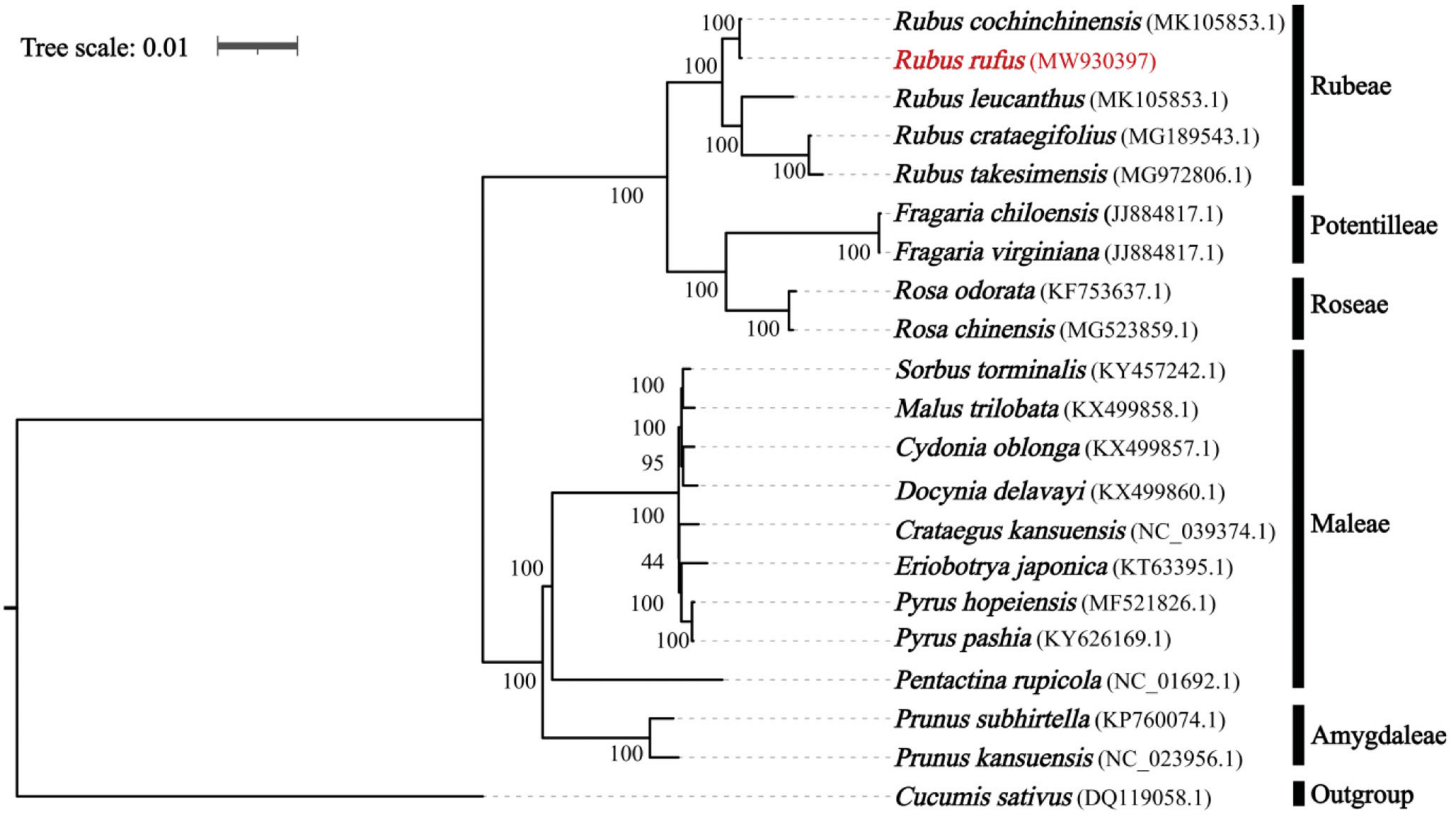
Maximum-likelihood tree of Rosaceae base on complete chloroplast genomes, with *Cucumis sativus* as outgroup. The *Rubus rufus* is marked in red and Bootstrap support values (based on 1000 replicates) are shown next to the nodes.

## Data Availability

The genome sequence data that support the findings of this study are openly available in GenBank of NCBI at https://www.ncbi.nlm.nih.gov/ under the accession no. MW930397. The associated BioProject, SRA, and Bio-Sample numbers are PRJNA739406, SRR14883822, and SAMN19789077, respectively.
